# Communicating a Congenital Cytomegalovirus Diagnosis: Parent Experiences and a Clinical Framework

**DOI:** 10.3390/ijns12030057

**Published:** 2026-07-24

**Authors:** Megan Honor Pesch, Laura C. Taylor, Sean P. McKenzie, Gail Demmler-Harrison

**Affiliations:** 1 Division of Developmental and Behavioral Pediatrics, Department of Pediatrics, University of Michigan, Ann Arbor, MI 48109, USA; 2 Division of Geriatric and Palliative Medicine, University of Michigan Health, Michigan Medicine, Ann Arbor, MI 48109, USA; taylorlc@umich.edu; 3 Medical Advisory Committee, National CMV Foundation, Tampa, FL 33612, USA; smckenzie81@gmail.com; 4 Division Pediatric Infectious Diseases, Department of Pediatrics, Baylor College of Medicine and Texas Children’s Hospital, Houston, TX 77057, USA; gdemmler@bcm.edu

**Keywords:** congenital cytomegalovirus, uncertainty, communication, physician-patient communication

## Abstract

Congenital cytomegalovirus (cCMV) is characterized by highly variable outcomes; how diagnostic information is communicated to families is critical, yet parent experiences of communication following a cCMV diagnosis are not well described. The objective was to examine parent experiences of communication following a cCMV diagnosis and identify opportunities to improve communication in the setting of clinical uncertainty. The method used was semi-structured interviews with 41 mothers of children with cCMV explored communication of diagnostic results, interactions with healthcare providers, and perceptions of information clarity, timing, and adequacy. Transcripts were analyzed using thematic analysis. Participants described variability in diagnostic communication, including differences in timing, modality, and content. Some received in-person counseling, whereas others learned of the diagnosis through phone calls, written communication, or electronic health portals prior to provider contact. Many reported limited explanation, insufficient anticipatory guidance, and inconsistent or incomplete information across providers. These communication gaps contributed to confusion, increased reliance on external information sources, and diminished trust in the healthcare system. Participants emphasized the importance of clear, compassionate communication that acknowledges uncertainty while providing actionable guidance. Communication surrounding cCMV diagnosis is inconsistent and often does not meet family needs. Diagnostic disclosure should function as a clinical intervention requiring clear, patient-centered, longitudinal communication to support families navigating uncertainty.

## 1. Introduction

Congenital cytomegalovirus (cCMV) is the most common congenital infection and a leading cause of childhood hearing loss and neurodevelopmental disability. Clinical presentation is highly variable, ranging from severe congenital disease to asymptomatic infection at birth, with sequelae that may emerge months or years later. This variability introduces substantial prognostic uncertainty, complicating both clinical management and communication with families [1,2,3,4].

In recent years, increasing attention has been directed toward earlier identification of cCMV through targeted or universal newborn screening [5]. Early diagnosis may facilitate access to antiviral therapy, early intervention services, and closer developmental monitoring [6]. However, earlier identification also increases the number of infants diagnosed in the newborn period without clear or immediate symptoms. In these settings, often in the hospital or early outpatient care, clinicians must communicate complex and evolving information to families navigating uncertainty at a particularly vulnerable time.

Communication in the context of uncertainty is inherently challenging [7]. Prior work in pediatrics has demonstrated that clinician communication of risk and prognosis influences parental understanding, emotional response, and decision-making. Effective communication requires not only accurate information but also clear framing of uncertainty and actionable guidance [8]. Despite this, approaches to communicating uncertainty remain inconsistent, and clinicians often lack structured strategies for discussing conditions with variable outcomes [8].

In cCMV, these challenges are particularly pronounced [6]. Diagnosis often occurs in the neonatal period or early infancy, when families are already navigating the emotional and logistical demands of a new child. At the same time, the information provided may be limited, evolving, or difficult to interpret, requiring families to process uncertainty while making decisions about treatment, monitoring, and engagement with care [4,9,10]. Inconsistent communication across providers and settings may further complicate this process, contributing to confusion and distress.

Understanding how families experience diagnostic communication in cCMV is essential to improving care. While prior studies have examined parental knowledge and awareness of cCMV, less is known about how diagnoses are communicated and how these interactions shape trust, understanding, and engagement with the healthcare system [4,6,10,11].

In this study, we examine parent experiences of communication following a diagnosis of cCMV. Using qualitative interviews with mothers of children diagnosed with cCMV, we explore how diagnostic information is delivered, how uncertainty is conveyed, and how communication practices influence parental interpretation and response. Our goal is to identify opportunities to improve communication in cCMV and to inform broader approaches to diagnostic disclosure in conditions characterized by uncertainty.

## 2. Methods

This study presents a qualitative analysis of in-depth interviews with mothers of children diagnosed with congenital cytomegalovirus (cCMV). The data were drawn from a broader qualitative study examining family experiences following a cCMV diagnosis, called the Mama Bears Study.

Participants were recruited through networks of families affected by cCMV, including clinical programs and advocacy organizations. Eligible participants were parents or primary caregivers of a child (now aged 3–15 years old) diagnosed with cCMV in early life, able to provide confirmatory testing results demonstrating that cCMV had been isolated by PCR from urine, serum or dried blood spot collected prior to 21 days of age, or via amniocentesis in utero. Eligible participants were also comfortable completing an interview and questionnaires in English and were aged 18 years or older. The study was approved by the University of Michigan Institutional Review Board. All participants provided informed consent and received a $40 gift card for completion of the semi-structured interview. A total of forty-two mothers responded, and forty-one mothers completed the study protocol (the one mother who did not participate had scheduling difficulties), reflecting the experiences of forty-one children with cCMV aged 3–15 years at the time of interview.

Questionnaire measures: Participants reported sociodemographic and birth history information via questionnaire.

Semi-structured interview: The COREQ (Consolidated Criteria for Reporting Qualitative Research) checklist (Supplemental Materials) is a 32-item checklist developed to improve the transparency and completeness of reporting qualitative studies, particularly those using interviews and focus groups [12]. Semi-structured interviews were completed by a trained qualitative researcher affiliated with the University of Michigan Survey Research Center. The interviewer held a master’s degree, had approximately five years of experience conducting qualitative research, and was not involved in the study authorship. No prior relationship existed between the interviewer and participants. At the beginning of each interview, the interviewer introduced themselves as a member of the research team interested in understanding the experiences of families affected by cCMV.

Each participant completed a single remote interview. Interviews were conducted with the interviewer in a private office setting, while participants joined from a location of their choosing. A semi-structured interview guide was used to facilitate discussion while allowing flexibility to explore topics raised by participants. Topics included experiences during pregnancy, the diagnostic process, communication with healthcare professionals, child development, access to medical and therapeutic services, and healthcare navigation. The interview guide was developed collaboratively by a multidisciplinary team that included a developmental and behavioral pediatrician, infectious disease physicians, and two mothers of children with cCMV who were not study participants. The interview guide is presented in Supplemental Materials. Interviews also examined participants’ emotional experiences, perceptions of uncertainty, and reflections on the long-term effects of receiving a cCMV diagnosis.

The flexible interview format encouraged participants to expand on issues they considered most important, generating detailed narrative accounts across a range of experiences. All interviews were audio-recorded, professionally transcribed verbatim, and reviewed to ensure transcription accuracy. Interview duration ranged from approximately 25 min to more than one hour and took place in 2024–2025. Ongoing discussions among the interviewer and study team were used to assess thematic saturation throughout data collection; however, recruitment continued until a total of 40 interviews had been completed.

Data Analysis: Data were analyzed using thematic analysis [13,14,15] with an inductive approach to identify patterns in parents’ experiences of communication surrounding their child’s cCMV diagnosis. The analysis was conducted by a master’s-level qualitative researcher with extensive formal training and experience in qualitative methods. To minimize the influence of pre-existing assumptions and clinical perspectives, the analyst was not involved in study design, development of the interview guide, participant recruitment, data collection, or manuscript authorship. The analyst was intentionally engaged as an independent contractor to enhance analytic neutrality and reduce potential bias, as such field notes from the interview were not used in the analysis.

Analysis began with repeated reading of interview transcripts to achieve immersion in the data and familiarity with participants’ narratives. An initial coding framework was developed inductively through close examination of the transcripts, allowing codes to emerge from the data rather than being derived from a predetermined theoretical framework. Early codes captured key aspects of communication experiences, including the timing of diagnosis, communication modality, information content, clarity of explanations, consistency of messaging across providers, perceptions of provider knowledge, emotional responses to communication encounters, and unmet informational needs.

Coding proceeded iteratively, with the coding framework refined as new concepts emerged and relationships between codes became apparent. The analyst used constant comparison techniques to examine similarities and differences both within and across interviews. Related codes were grouped into broader categories and subsequently organized into themes that reflected common patterns in participants’ experiences. Attention was paid to identifying communication practices that facilitated or hindered understanding, trust in healthcare providers, engagement with recommended care, and adaptation to prognostic uncertainty.

Throughout the analytic process, emerging codes and themes were discussed with members of the research team to ensure interpretive rigor and to challenge assumptions. Disagreements regarding interpretation were resolved through discussion and review of the original transcripts. Themes were repeatedly examined against the source data to ensure they accurately reflected participants’ narratives and remained grounded in the interview content. The analysis sought to preserve variation in individual experiences while identifying overarching patterns across participants. A participant check of the final codes was conducted with two parents of children with cCMV. Representative quotations were selected to illustrate major themes and provide evidence supporting analytic interpretations.

Communication framework development: Following completion of thematic analysis, the research team developed a communication framework to translate the study findings into practical recommendations for clinicians communicating a cCMV diagnosis. Framework development was informed by the major themes identified across interviews, with an emphasis on communication practices consistently described by participants as helpful or associated with improved diagnostic experiences. The framework was refined through iterative discussions among the multidisciplinary research team to ensure that recommendations aligned with the qualitative data and were feasible for clinical implementation. To enhance relevance and usability, the draft framework was subsequently reviewed by a group of mothers of children with cCMV who provided feedback regarding its clarity, completeness, and applicability to the diagnostic experience. Participants completed a brief semi-structured interview regarding whether the framework reflected their experiences, whether important elements were missing, and whether the recommendations were feasible for healthcare providers. Feedback was analyzed using rapid qualitative content analysis and incorporated into the final framework.

## 3. Results

### 3.1. Cohort Characteristics

A total of forty-one mothers of children with congenital cytomegalovirus (cCMV) participated in the study. Mothers had a mean age of 37.4 years (SD = 6.2). Most identified as non-Hispanic White (85.4%), while 12.2% identified as another racial or ethnic group, including Black non-Hispanic, Asian, and Hispanic participants. The cohort was highly educated, with 65.8% of mothers holding a bachelor’s degree or higher and 26.8% reporting a master’s or doctoral degree.

Children were a mean of 7.5 years old (SD = 3.4) at the time of interview. Approximately one-fifth of children (19.5%) were diagnosed with cCMV prenatally, while most were diagnosed during early infancy, with 48.7% identified before 2 months of age and 31.7% diagnosed at 2 months of age or later. Over half of children were female. At birth, children represented a range of cCMV presentations, including symptomatic disease, asymptomatic infection with isolated sensorineural hearing loss, and asymptomatic infection.

Based on maternal report, the most recalled manifestations of cCMV at birth were small for gestational age status (68.3%), microcephaly (61.0%), abnormalities on neuroimaging (61.0%), and hearing loss (58.5%). Other reported findings included petechiae (36.6%), hepatosplenomegaly (29.3%), and chorioretinitis (7.3%). These characteristics reflect a cohort with substantial representation of children who experienced clinically significant manifestations of cCMV during infancy.

In terms of which type of providers was most likely to deliver the diagnosis, Neonatologists or Newborn hospitalists most often gave the diagnosis (n = 11), followed by General Pediatricians (n = 4) and Neurologists (n = 4) (Table 1). Results were most often delivered in a face-to-face conversation with a physician (n = 21), or by phone (n = 9), whereas four mothers received the information through auto-released results in the electronic medical record. Data demonstrating provider type by format by which the diagnosis was delivered, and by quality of diagnosis delivery are presented in Supplemental Table S1a,b. Positive aspects of the cCMV diagnosis disclosure are presented in Supplemental Table S1c.

### 3.2. Thematic Overview

Participants described substantial variability in how cCMV diagnoses were communicated, with differences in timing, modality, content, and coordination across providers. Communication was often experienced as fragmented, incomplete, or difficult to interpret, particularly given the inherent uncertainty of cCMV. These experiences shaped mothers’ understanding of the diagnosis, engagement with care, and trust in the healthcare system. Five major themes emerged: (1) variability in diagnostic disclosure, (2) gaps in explanation and anticipatory guidance, (3) inconsistency and lack of coordination across providers, (4) reliance on external information sources, and (5) the importance of compassionate and direct communication. Illustrative quotes from each theme are presented in Table 2.

#### 3.2.1. Variability in Diagnostic Disclosure

Mothers described a wide range of experiences in how they first learned of their child’s diagnosis. Some received in-person counseling, often in hospital settings or during clinical visits. Others were informed by phone, sometimes with limited opportunity for immediate follow-up. A few learned of the diagnosis through written communication or by viewing results in the electronic health record before speaking with a clinician.

Receiving the diagnosis outside of a direct conversation with a provider was described as particularly distressing due to a lack of immediate context or support, contributing to confusion and heightened emotional responses. Even among those who received in-person disclosure, the timing and structure of communication varied considerably, with some describing clear and supportive conversations and others reporting brief or fragmented interactions.

#### 3.2.2. Gaps in Explanation and Limited Anticipatory Guidance

Through interviews, many mothers described receiving limited information about what cCMV is and its implications for their child. Information provided at diagnosis was often described as minimal, overly general, or focused on worst-case scenarios without contextualization.

Participants frequently reported a lack of anticipatory guidance regarding the range of possible outcomes, what to monitor over time, and next steps in care. Without clear guidance, parents were left to interpret complex and uncertain information on their own. This contributed to difficulty understanding the diagnosis and heightened anxiety, particularly in the early period following disclosure.

Several participants noted that providers appeared uncertain themselves or were unable to answer questions. Some physicians seemed unfamiliar with cCMV in general, reinforcing the perception that information was incomplete or unreliable and contributing to uncertainty, diminished trust in the healthcare system, and a perceived need to seek out additional information independently.

#### 3.2.3. Inconsistency and Lack of Coordination Across Providers

Many mothers described receiving inconsistent or conflicting information from different providers. Variability in messaging regarding prognosis, recommended evaluations, and next steps contributed to confusion and, in some cases, frustration.

Participants also described a lack of coordination across members of the care team, with some reporting that providers assumed others had already communicated key information. In a few cases, parents learned new or contradictory information at subsequent visits, which they described as a “moving target” rather than a coherent care plan.

These experiences were often interpreted as reflecting gaps in provider knowledge or system-level coordination, and in some cases contributed to diminished trust in the healthcare system.

#### 3.2.4. Reliance on External Information Sources

In response to gaps in communication, many mothers described seeking information independently. Despite being advised by some providers not to search online, participants frequently turned to the internet, social media, and other families for information about cCMV.

While these sources sometimes provided helpful context and connection, they also introduced additional challenges. Some mothers described their partners or other family members as feeling more emotionally able to research cCMV and navigate available resources during the early stages after diagnosis. The wide range of possible outcomes described online often amplified uncertainty and anxiety. Participants described difficulty distinguishing relevant or accurate information, particularly when trying to apply general information to their child’s specific situation. The central challenge was not access to information alone, but the lack of interpretive structure needed to translate complex or uncertain information into informed decision-making.

This reliance on external sources reflected both an unmet need for additional information and a lack of clear, consistent, and trusted clinical guidance.

#### 3.2.5. The Importance of Compassionate and Direct Communication

Despite variability in experiences, participants were clear about what they found helpful in communication. Mothers emphasized the importance of receiving information directly from a knowledgeable provider in a timely and compassionate manner.

Several mothers reported that their anxiety decreased substantially after connecting with a healthcare provider who had expertise in cCMV, particularly following consultations with providers who had limited familiarity with the condition. Two mothers noted that primary care pediatricians had limited knowledge about cCMV when giving the diagnosis, but their promise to learn about the disorder and quickly follow-up with the family was appreciated. Importantly, many mothers did not seem to expect their provider to have all the answers. Rather, participants valued communication that acknowledged uncertainty while still providing clear explanations and concrete next steps. Several mothers mentioned specific providers, whether they were a primary care provider or an infectious disease specialist, who were honest about the uncertainty inherent in the diagnosis, while also explaining what was known. Rather than avoiding difficult information, parents preferred transparency paired with known facts and support. Communication that emphasized the range of possible outcomes, without overemphasizing worst-case outcomes, was described as particularly helpful. Some mothers described that meeting with a specialist who was able to share knowledge about cCMV in general was helpful, but that person was not always the same person who gave the diagnosis.

Positive communication experiences were associated with greater confidence in the care team and a stronger sense of partnership in managing the child’s condition. Coordinated referrals and clear next steps, both about the clinical evaluation, and what to look out for in terms of child development were specifically mentioned by some mothers as helpful. In contrast, poor communication, whether due to timing, delivery, or content, negatively affected trust and engagement with care.

Conceptual Model: Based on these findings, we propose a clinical communication framework (CLEAR: Clarity, Listening, Expectation-setting, Action, Revisit) to guide diagnostic disclosure in cCMV and similar conditions (Table 3). CLEAR was developed through an iterative process in which themes identified from parent interviews were mapped onto established evidence-based communication frameworks, such as the SPIKES protocol [16,17], VitalTalk [18], Ask-tell-ask [19], and the Serious Illness Conversation Guide [20]. These existing models emphasize delivering serious news with empathy, eliciting patient, and family perspectives, and engaging in shared decision-making. However, they were primarily developed for conversations in which clinicians possess greater prognostic certainty or where treatment decisions occur over a relatively defined period. In contrast, a diagnosis of cCMV frequently introduces prolonged uncertainty regarding developmental, neurologic, and hearing outcomes, requiring communication that extends well beyond the initial disclosure. CLEAR therefore preserves the core strengths of these established approaches while explicitly emphasizing expectation-setting around uncertainty, providing concrete action steps despite incomplete prognostic information, and intentionally revisiting conversations over time as new information emerges by explicitly incorporating strategies for communicating uncertainty and supporting families longitudinally (Table 4).

Three mothers participated in the review of the CLEAR framework. Overall, participants described the framework reflecting their diagnostic experiences and endorsed its emphasis on empathy, transparent communication, acknowledgment of uncertainty, and provision of clear next steps. Participants emphasized that compassionate communication should be paired with actionable guidance and access to knowledgeable providers. Minor revisions were made to clarify wording and strengthen recommendations regarding follow-up support and information resources.

## 4. Discussion

In this qualitative study of mothers of children diagnosed with congenital cytomegalovirus (cCMV), communication emerged as a central determinant of how families understood and responded to diagnosis. Participants described substantial variability in how diagnostic information was delivered, including differences in timing, modality, content, and coordination across providers, which shaped parents’ understanding of cCMV, their emotional responses, and their trust in the healthcare system. Taken together, these findings suggest that diagnostic communication is not the neutral transfer of information, but a critical clinical intervention, particularly in conditions characterized by uncertainty.

Participants described a wide range of communication experiences, including learning of the diagnosis through electronic health portals prior to clinician contact, receiving minimal explanation at the time of disclosure, and encountering inconsistent messaging across providers. These findings reflect broader challenges in communicating complex and uncertain diagnoses, particularly in the neonatal period, when care is often fragmented across multiple providers and settings [7].

Gaps in anticipatory guidance were especially salient. Many mothers reported receiving little information about what to expect, what to monitor, or how to interpret evolving findings. In the absence of structured guidance, parents were left to construct their own understanding, often turning to online sources that amplified rather than clarified uncertainty. These experiences contributed to confusion and diminished trust in the healthcare system. With increasing cCMV screening over the last several years [5], it is possible that anticipatory guidance may be improving through the safety net provided by health department run programs.

A key finding of this study is that diagnostic communication in cCMV is an ongoing process that unfolds as children develop and new information emerges rather than a single event. Families described repeatedly revisiting the diagnosis, reinterpreting its meaning, and adjusting expectations over time. Despite this, communication practices often remained anchored to the moment of diagnosis. Participants described limited follow-up discussions and a lack of continuity regarding how information was revisited or updated. This mismatch between the longitudinal nature of uncertainty and the episodic nature of communication represents a critical gap in care. These findings support a shift in how diagnostic communication is conceptualized, from a discrete event to an ongoing clinical responsibility.

### Implications for Clinical Practice and Screening Programs

A key contribution of this work is the development of the CLEAR framework, a family-informed communication framework designed to guide clinicians delivering a cCMV diagnosis. The framework emphasizes several key principles. First, clinicians should prioritize clarity, using accessible language to ensure that families understand the diagnosis. Second, communication should include explicit acknowledgment of emotional responses, creating space for parents to process information. Third, uncertainty should be named directly and framed to convey the full range of possible outcomes. Fourth, families should be provided with actionable next steps, including plans for monitoring and intervention. Finally, communication should be revisited as new information emerges, recognizing that understanding and clinical needs evolve over time. Importantly, this framework positions communication not as the transfer of information, but as a clinical intervention that shapes how families understand and respond to uncertainty.

Although developed from parent experiences of cCMV, the communication principles reflected in the CLEAR framework are consistent with approaches recommended in other clinical contexts in which families must navigate uncertainty following newborn screening or diagnostic testing. In newborn screening, conditions such as cystic fibrosis screen-positive, inconclusive diagnosis (CFSPID/CRMS) and spinal muscular atrophy have highlighted the importance of communication strategies that emphasize transparency, empathy, clear next steps, and longitudinal follow-up. Similarly, counseling surrounding variants of uncertain significance (VUS) identified through genetic testing requires clinicians to communicate incomplete information while maintaining trust, supporting informed decision-making, and avoiding both false reassurance and unnecessary alarm. Although the source of uncertainty differs, a confirmed diagnosis with uncertain prognosis in cCMV versus uncertainty regarding the clinical significance of a genetic finding, the communication principles are remarkably consistent. This convergence across pediatric medicine supports the broader applicability of these communication principles while underscoring the need for cCMV-specific guidance. Rather than representing a new communication paradigm, CLEAR is intended as an adaptation of established serious illness communication frameworks for the unique longitudinal uncertainty that characterizes cCMV and other pediatric conditions with evolving prognoses.

An additional consideration is the increasing availability of laboratory results through electronic patient portals following implementation of the federal information-blocking provisions of the 21st Century Cures Act. Although timely access to health information promotes transparency and patient autonomy, several participants in our study learned of their child’s cCMV diagnosis through the electronic health record before speaking with a clinician. For unexpected or emotionally significant diagnoses, receiving results without clinical context may heighten confusion and distress. These findings underscore the importance of systems that facilitate prompt clinician follow-up after result release and prepare families for the possibility of receiving diagnostic information electronically.

Concerns about uncertainty are often cited as a potential harm of screening [21]. Our findings suggest that uncertainty alone is not the primary driver of distress. Rather, distress arises when uncertainty is not accompanied by clear explanation, guidance, and support. This distinction underscores the importance of communication in realizing the benefits of early identification. Prior work has shown that if a general anticipatory roadmap is provided with clear communication and expertise, families can tolerate or even embrace the ambiguity that is inherent with cCMV [4,9]. Screening programs should therefore incorporate structured communication strategies, including clinician training, standardized counseling approaches, and systems for longitudinal follow-up [22]. Without these supports, expanded identification risks increasing informational burden without improving family experience or outcomes.

This study provides in-depth insight into parent experiences of diagnostic communication in cCMV, drawing on a large qualitative sample and capturing a range of perspectives across different stages of childhood. The qualitative design allowed for exploration of nuanced experiences that are not readily captured through quantitative measures.

Several limitations should be considered. The study reflects maternal perspectives and may not capture the experiences of other caregivers or fathers. Future research should include multiple caregivers to provide a more comprehensive understanding of family experiences following a cCMV diagnosis. Participants were recruited through clinical and advocacy networks, which may limit generalizability to families with less access to care. Additionally, because participation was voluntary, families with particularly memorable, whether positive or negative, diagnostic experiences may have been more likely to participate, potentially influencing the range and distribution of perspectives represented in the sample. Retrospective accounts may be influenced by time since diagnosis and subsequent experiences with the healthcare system.

As newborn screening for cCMV expands, communication will become an increasingly important component of clinical care. This study suggests that uncertainty need not be viewed as unavoidable harm of diagnosis, but rather as a challenge that can be meaningfully addressed through thoughtful, compassionate, and longitudinal communication. By framing communication as a clinical intervention rather than simply the delivery of information, the CLEAR framework offers a practical, family-informed approach to supporting parents through the evolving journey of cCMV.

## 5. Conclusions

Communication following a diagnosis of congenital cytomegalovirus is variable, often insufficient, and highly consequential. In conditions characterized by uncertainty, the way information is communicated shapes not only parental understanding but also emotional response, trust, and engagement with care.

These findings suggest that the central challenge in cCMV is not whether uncertainty can be eliminated, but how it is conveyed and supported. Diagnostic communication should be understood as an ongoing clinical process and a critical intervention. Efforts to expand cCMV screening must therefore be accompanied by parallel investments in communication infrastructure, ensuring that families are informed and supported in making sense of what remains unknown.

## Figures and Tables

**Table 1:** Participant characteristics (N = 41 mothers and 41 children with congenital cytomegalovirus).

Characteristic		N or Mean	% or SD
Maternal age (years)		37.4	6.2
Maternal race/ethnicity	White NH	35	85.4
	Other (Black non-Hispanic, Asian, White Hispanic)	5	12.2
Highest level of maternal education	High school diploma or less	3	7.3
	Some college/associate’s degree/Technical certificate	10	24.4
	Bachelor’s degree	16	39.0
	Masters or Doctoral degree	11	26.8
Child sex	Female	22	53.7
Child age at time of interview (years)		7.5	3.4
Age at diagnosis of cCMV	In pregnancy	8	19.5
	From birth to <2 months	20	48.3
	2 months or older	13	31.7
cCMV severity at birth *	Symptomatic	31	75.6
	Asymptomatic with isolated SNHL	6	14.6
	Asymptomatic	4	9.8
Symptoms present at birth (select all that apply)	Small for gestational age	28	68.3
	Microcephaly	25	61.0
	Findings on brain imaging	25	61.0
	Petechiae	15	36.6
	Chorioretinitis	3	7.3
	Hepatosplenomegaly	12	29.3
	Hearing loss	24	58.5
	None	4	9.8

* Based on maternal recollection of signs, symptoms and findings.

**Table 2:** Themes and illustrative quotes from interviews with mothers of children with congenital cytomegalovirus.

Themes and Representative Quotes
**1. Variability in Diagnostic Disclosure**
*“So, this is something that’s really unsettling for me… when it comes to technology and the medical field. I learned about his CMV diagnosis because, they posted the results of his MRI on our portal. So I was able to access it before anybody called me, and it was after hours, so I couldn’t get ahold of a doctor. I was reading through his MRI report, and I saw all of this information. And I had no idea what it meant. So, I started googling everything. And was spiraling down a rabbit hole of all these different things that could possibly happen with CMV. Because it’s such… kids are affected so differently. I had no access to a doctor or anybody to talk to, because they posted the results on our portal through our children’s hospital before ever getting a phone call or anything from any doctor or technician. It was incredibly stressful. We had no idea what was going on. I can’t remember exactly, like, if it was one day or two days, but eventually we did receive. A phone call… Parents should be reached out to. Whether it’s them or their kids, by a human being before posting anything on there. On their medical page. I was really upset about. I said, that’s not…. That’s not right. You shouldn’t be doing that to people”.*—Participant 35
*“Well, she tested positive while we were still in the hospital. So… (the) neurology and infectious disease residents came in and told us and then the neonatologist came in. They had all discussed it. One of the residents came in the middle of the night at like 2 a.m. And told me that it was positive. Probably wasn’t the best option for them to do that, especially not without their attending with them. So that was kind of frustrating because they had kind of given grim hope that she had all these signs. And basically, there’s nothing we can do… (they) pretty much said ‘take her home.’”*—Participant 13
**2. Gaps in Explanation and Limited Anticipatory Guidance**
*“(Learning about her cCMV) Yeah, it was awful. It was the worst. It was the worst time of my life. Every day you just, you know, there wasn’t a lot of hope from the first perinatal team that I saw. There wasn’t a lot of information as far as what things could look like for (her). Nobody knew nobody could tell me anything.”*—Participant 12
*“I didn’t even really understand it then. It was… because I’d never heard of it. And they tried to explain it to me, and I’m just like, um, not really understanding it. Now I know that it’s a strain of herpes virus, but it’s like, you don’t have herpes, it’s like cold sore herpes virus, not like… sexual herpes virus. Like, and I was just learning that last year.”*—Participant 40
**3. Inconsistency and Lack of Coordination Across Providers**
*“We were almost too late to start the medication. It was one of the most stressful medical experiences I’ve had, the pediatrician’s office was like, ‘ (the infectious disease doctor) will talk with, you know, we’ll see if she thinks it’s appropriate to prescribe this medication And if she does, then we need to start it as soon as possible’. So, I’m calling that infectious disease doctor’s office saying like, “I need to follow up about this medication, and the front desk just keeps telling me like,’ you have an appointment in four weeks. We can talk about it then.’ And I’m like, no, we can’t! I need, I need… this now and I’m calling and I think I called several times to the point where the last time I called, I’m in tears and I’m like ‘can I please just get this message there’s a misunderstanding here. I’m just trying to get it cleared up.’ And I think once you have a mom on the phone that’s crying, the front desk gets you through.”*—Participant 18
*“Um, she [ob/gyn]… she said she had her suspicions on it. We wouldn’t know until, uh, the test results came back, but he did have. Many symptoms of cCMV. And, um…. She…. She got really mad at me because I wanted to be transferred to a children’s hospital instead of a…. You know, a typical hospital that you go to in any of the cities in [STATE] And she said that they did a head ultrasound, and it was… it showed some calcifications, but they were not going to do an MRI. Um, she said that if I was transferred to Children’s, that they would. They would do the exact same thing that she would be doing. And, um, she told me that they probably wouldn’t even accept him at Children’s Hospital. Uh, she called them while we were staying about 5 feet away from her, and they told her, we’re on our way to get him, and brought an ambulance, and came and picked him up, and transferred him to Children’s Hospital. Um, when we got to Children’s Hospital, they immediately started all the testing. They… we had to do a second round of platelet transfusion They did MRI, um… we did the hearing screening. He did not have hearing loss at the time. Um, but children’s… it was just…. 100 times better. They had seen many kids with cCMV, uh, while [child] did not. And so the doctor was just very rude about it and acted like there was nothing we could do or no further testing. And that we were just gonna go home. But that changed.*”—Participant 39
**4. Reliance on External Information Sources**
*“There’s a lot of families that have children with way worse medical situations, very unstable health wise for their kids or families and I’m connected to a Facebook group for families of CMV. I had to unfollow the group because it was kind of just seeing all that was very um when you see somebody who says, oh, my baby didn’t make it, you know, we were this far along, it’s because of CMV or things like that. It’s really hard and it’s really it’s hard not to in a way you just you’re so grateful that she’s so healthy and doing okay, but then you just feel so terrible because that could have been it could have been her.”*—Participant 12
*“I found that CMV moms’ community on Facebook, that group. I found them while we were still in the NICU, and that group was… so instrumental in just helping me navigate, okay, what do I do? Okay, Social Security. Get her on disability, make sure she’s got insurance, all of the things. They were really instrumental in giving me advice in how to um… get those things taken care of, but then also just a space to vent, to… to grieve the… the life that I had envisioned while I was pregnant. And… once I had grieved all that, then I realized, like, okay, well, the doctors were wrong.”*—Participant 31
*“That’s just how it felt at that time. Very misunderstood. I didn’t get it until later. I really just didn’t understand it, and I did my own research, and I got more out of researching myself than I did from the actual doctor telling me, and nurses telling me, because I really think that they didn’t even really, They hadn’t really seen cases of (cCMV). It’s common, but it’s not, like… So, yeah. It’s a shame that… the burden of education falls on… the mother.”*—Participant 40
**5. The Importance of Compassionate and Direct Communication**
*“So, our pediatrician, we have a wonderful pediatrician. And because [STATE]’s small state by population, the results had to be sent out. The blood draw or whatever. I think it was a urine sample had to be sent out. And so, it took a few days to come back and [DOCTOR], he called himself and said, you know. The results were positive And so we’d like to have [CHILD] come in as soon as possible and talk about what to do.”*—Participant 18
*“He [GI specialist] called me one day and told me that her urine test came back and that she was positive for CMV. And that she most likely got it from me when I was pregnant and that basically all he told me and then left it at that. And I’m like, what the heck does that mean? You know, and you’re just like Okay, so then of course I googled And I did learn a lot, but then I basically was going worst case scenario with everything. So when I was looking through everything, I’m sitting here and I’m reading you know, I’m reading these stories about kids that have CMV that are wheelchair bound that are that are tube fed and that are, you know, 100% dependent on somebody, right? And I’m just so I called my pediatrician up. And I’ve been with this pediatrician ever since and I will not change to a different doctor because even when she, you know, when I called her up and I told her and she was and I was hysterical in tears and I was like, I don’t know what this means, it’s all he told me. She was like, I don’t have the answers for you. But I know who will. She’s like give me an hour…”*—Participant 7

**Table 3:** The CLEAR Framework for congenital CMV diagnosis disclosure.

Domain	Clinical Goal	Recommended Practices	Example Language
C—Clarity of Diagnosis	Ensure parents understand what cCMV is	• Use plain language	
• Avoid jargon	Share the “headline” of this new diagnosis—what it is AND what does it mean for the child		“Your child has a viral infection called CMV that can affect development, but it looks different in every child.”
L—Listening & Emotional Acknowledgment	Recognize emotional impact	• Pause after disclosure	
• Name emotions	“I know this may be shocking news to hear.” “I wish that I had different news to share today.”		
• Validate reactions	“I know this is a lot to take in. Many parents feel overwhelmed when they hear this.”		After this step, it can be helpful to ask permission to move forward in the conversation. “May I share with you more information about this condition that I find many parents like to know?” Then you can delve into the piece about uncertainty in prognosis and effects.
E—Expectation-Setting for Uncertainty	Normalize uncertainty without minimizing it	• Explicitly name uncertainty	
• Frame range of outcomes			
• Avoid definitive predictions	“We don’t know exactly how this will affect your child. Some children have mild effects; others need more support.”		After going into the uncertainty regarding possible future effects, recommend pausing for questions. “I know I’ve shared a lot of information. What questions do you have so far about what we’ve discussed?” Spend time answering questions and clarifying information.
A—Actionable Next Steps	Provide direction despite uncertainty	• Give concrete plan	Ask permission to move forward through each step. “May I share with you my thoughts about specific next steps in your child’s care?”
• Outline monitoring			
• Connect to services	“Here’s what we’ll do next—we’ll check hearing regularly and connect you with early intervention.”		
R—Revisit & Longitudinal Support	Reinforce that communication is ongoing	• Schedule follow-up	
• Re-explain over time	Ensure parents understand what cCMV is and how it may impact their child		
• Provide resources	“We’ll keep coming back to this together as your child grows and we learn more.”		

**Table 4:** Comparison of Communication Frameworks for Diagnosis in Conditions with Uncertainty.

		Communication Frameworks		
Features	SPIKES [18]	Serious Illness Guide [21]	VitalTalk [19]	CLEAR (Proposed)
Primary Focus	Breaking bad news	Goals of care	Serious illness communication	Uncertainty in outcomes/prognosis
Emotional Support	Yes	Yes	Yes	Yes (explicit emphasis)
Handles Uncertainty	Limited	Partial	Yes	Central focus
Provides Actionable Steps	Some	Yes	Yes	Strong emphasis
Longitudinal Approach	No	Limited	No	Yes (Revisit step)
Designed for NBS/CMV	No	No	No	Yes (adapted)

Abbreviations: CMV:, cytomegalovirus; NBS:, newborn screening.

## Data Availability

Limited deidentified qualitive data is available from the authors upon request in accordance with the informed consent. Full quantitative data is also available from the authors upon request.

## Supplementary Materials

The following supporting information can be downloaded at: https://www.mdpi.com/article/10.3390/ijns12030057/s1, COREQ Checklist; Mama Bears Semi-Structured Interview Guide; Table S1a: Provider type by format in which the diagnosis of cCMV was delivered to the family; Table S1b: Quality of the delivery the diagnosis of congenital cytomegalovirus by type of healthcare provider; Table S1c: Positive aspects of cCMV diagnosis disclosure mentioned in participant interviews.
