# Supplementary material for: Communicating a Congenital Cytomegalovirus Diagnosis: Parent Experiences and a Clinical Framework
Source: Int J Neonatal Screen. 2026 Jul 24;12(3):57. doi: 10.3390/ijns12030057 (PMC13510796; doi:10.3390/ijns12030057)
Supplement: Supplementary file 1 [file IJNS-12-00057-s001.zip › IJNS-4389520-supplementary.pdf]

Supplemental Materials

COREQ Checklist (32 Items)

**Item**	**Guide Question/Description**	**Reported?**	**Location in Manuscript**	**Page**
**Domain 1: Research Team and Reflexivity**				
1	Interviewer/facilitator	Yes	Methods, Semi-structured interview	6
2	Credentials	Yes	Master's degree; ~5 years qualitative research experience	6
3	Occupation	Yes	Qualitative researcher affiliated with University of Michigan Survey Research Center	6
4	Gender	No	NA	NA
5	Experience and training	Yes	Methods, Semi-structured interview	
6	Relationship established	Yes	"No prior relationship existed"	
7	Participant knowledge of interviewer	Yes	Interviewer introduced as member of research team	6
8	Interviewer characteristics	Yes	Analyst independence described	7
**Domain 2: Study Design**				
**Theoretical framework**				
9	Methodological orientation	Yes	Thematic analysis with inductive approach	
**Participant selection**				
10	Sampling	Yes	Purposive recruitment through clinical and advocacy networks	6
11	Method of approach	Yes	Recruitment sources described	6
12	Sample size	Yes	N = 41 mothers	6
13	Non-participation	Yes	Refusals/dropouts not reported	6
**Setting**				

Supplemental Materials

14	Setting of data collection	Yes	Remote interviews; interviewer in private office; participants in location of choice	6
15	Presence of non-participants	No	Not reported	NA
16	Description of sample	Yes	Table 1; cohort characteristics	Table 1 and page 9
Data collection				
17	Interview guide	Yes	Semi-structured guide; in Supplement	Sup. Mat.
18	Repeat interviews	Yes	Single interview per participant	6
19	Audio/visual recording	Yes	Audio recorded	7
20	Field notes	Yes	Not used due to bias	7
21	Duration	Yes	25 minutes to >1 hour	7
22	Data saturation	Yes	Discussed explicitly	7
23	Transcripts returned to participants	No	Not reported	NA
**Domain 3: Analysis and Findings**				
**Data analysis**				
24	Number of data coders	Yes	There was one analyst	8
25	Description of coding tree	Partial	Coding process described; coding tree not provided as this was not generated	8
26	Derivation of themes	Yes	Inductive; themes emerged from data	8
27	Software	No	Not reported – not used	NA
28	Participant checking	Yes	Member checking not reported	8
Reporting				
29	Quotations presented	Yes	Table 2	Table 2
30	Data and findings consistent	Yes	Results supported by quotations	Table 2
31	Clarity of major themes	Yes	Five themes clearly presented	Results and Table 2
32	Clarity of minor themes	Partial	Variation acknowledged but minor/disconfirming themes not extensively discussed	Throughout

Supplemental Materials

Mama Bears Semi-Structured Interview Guide

Alright [Participant name], ready to get started? I am going to ask you some questions about your experiences that I hope will generate a good discussion. There are no right or wrong answers. This interview will be recorded and transcribed and all identifying features, like your name and your child's name will be removed from the transcript for privacy reasons. At any point you can pass on questions or stop the interview. This should take about an hour. This should feel like a casual conversation, again however you answer the questions is fine. Any questions?

Okay. I'm going to start recording now [press record]

OK, let's get started. So again, my name is __________, and I am working with a team of researchers learning about the experiences of caregivers of children born with congenital CMV. For the record, my name is XXX and I am here with participant #####.

1. Great. Remind me, your child with congenital CMV, what is their name and how old are they now?

2. Aww, I always like to get a sense of what the kids are like before we get going. What kinds of things are he/she into right now? (Optional prompt if clarification needed – Like what do they like to play with, what interests do they have, things like that)

3. And how old was (child's name) when they were diagnosed with congenital CMV?

4. When was the first time you learned about cytomegalovirus or CMV?

Prompt. Now, I want to go back to the beginning of your -your partner's pregnancy with (child) and I'm going to ask you some questions in chronological order.

5. Tell me about your/your partner's pregnancy with (baby's name). Prompt Were there any medical issues or concerns that came up during the pregnancy? Prompt Was anything concerning ever mentioned to you or your partner about the ultrasounds?

6. Tell me about your or your partner's labor and delivery with _____.

7. And how did he/she do after she/he was born? (Optional prompts Like did she/he cry right away, how did he/she eat/nurse, any issues with the hearing screen, or baby's size?

8. **If congenital CMV was diagnosed in-utero, or as a newborn** (If diagnosed later proceed to question 9)

Supplemental Materials

a. Now you said that (child) was diagnosed with congenital CMV as an infant. Can you tell me why (child name)'s doctors tested (him/her) for congenital CMV? (Optional prompt – Sometimes babies are born in a state that tests everyone for congenital CMV, or other times children get tested because they might have hearing loss, or something babies can have certain features at birth like having a smaller head size, things like that).

b. How did you learn the results from (child)'s CMV testing? Did a healthcare provider tell you, did you get a letter etc.)

c. What was that like for you and your partner as new parents? Do you remember what you felt during those first weeks and months after getting the diagnosis?

d. Tell me about what happened next for (child) – did she/he get additional tests or see other specialists?

e. How did your family manage those extra tests and appointments? What about any appointments now?

f. How was (child name)'s health as an infant? And as a toddler? What about now?

g. Were there any concerns about (child name)'s development as an infant? Or toddler? What about at preschool age? What about now?

h. How do you think knowing that (child) had congenital CMV as an infant impacted their health? Access to resources? What about your relationship with him/her?

9. **If congenital CMV was diagnosed after 2 months old**

a. How was (child name)'s health as an infant? And as a toddler? What about now?

b. Were there any concerns about (child name)'s development as an infant? Or toddler? What about at preschool age? What about now?

c. Now you said that (child) was diagnosed with congenital CMV when he/she was xxxxx. Can you tell me why (child name)'s doctors tested (him/her) for congenital CMV? (Optional prompt – Sometimes children get tested because they might have hearing loss, or developmental delays).

d. How did you learn the results from (child)'s CMV testing? Did a healthcare provider tell you, did you get a letter, etc)

Supplemental Materials

e. What was that like for you and your family emotionally? Do you remember what you felt during those first weeks and months after getting the diagnosis?

f. Tell me about what happened next for (child) – did she/he get additional tests or see other specialists?

g. How did your family manage those extra tests and appointments? What about any appointments now?

h. How do you think finding out that child had congenital CMV when they were (xxx years/months old) impact their health? Access to resources? What about your relationship with him/her?

10. Looking back, how might have things been different if the doctors never tested (child) for congenital CMV? Prompts. What may have been different if they tested (child earlier)? What about later, say as an older child?

11. How do you think knowing about _____'s congenital CMV may impact his/her future? Like, the knowledge that she/he has CMV, not just the disease itself.

12. Overall, how do you feel about knowing (child) has congenital CMV? (Optional prompt, like are you glad to know, or is the knowledge a burden?)

13. Does ____ receive any early intervention or special education services or therapies? (Optional prompt why or why not? Can you tell me more about those services?)

14. What are you worried about for (child)'s future (Prompt – it can be anything, or nothing!) or with regard to your child's future, what do you think about?

15. Is there anything else you would like me to know, or that we didn't get to?

Supplemental Materials

Supplemental Table S1a. Provider type by format in which the diagnosis of cCMV was delivered to the family

**Provider type**	**Face-to-face**	**Phone**	**Other/ Written/ Unclear**	**Total**
Neonatologist / Newborn hospitalist	11	0	0	11
Neurologist	1	3	0	4
General pediatrician	1	3	0	4
MFM	3	0	1	4
ID physician	1	2	0	3
Other	2	1	2	5
Nurse in hospital	1	0	1	2
Unclear	1	0	7	8
	**21**	**9**	**11**	**41**

Supplemental Table S1b. Quality of the delivery the diagnosis of congenital cytomegalovirus by type of healthcare provider (N=30)*

**Provider type**	**Well**	**Both Good & Bad aspects**	**Poorly / Could be Improved**	**Details Not Shared / Unclear**	**Total**
Neonatologist/ Newborn hospitalist	0	1	3	7	11
Neurologist	0	0	4	0	4
General pediatrician	2	0	1	1	4
MFM	1	0	1	1	3
ID physician	1	0	2	0	3
Other	2	0	1	0	3
Nurse in hospital	0	0	1	0	1
Unclear provider	0	0	0	1	1
Total	**6**	**1**	**13**	**10**	**30**
* limited to those who received a congenital CMV diagnosis in person or by phone					

Supplemental Table S1c. Positive aspects of cCMV diagnosis disclosure mentioned in participant interviews (N=41):

**Select all that apply**	N	(%)
Kindness and compassion	2	4.9
Gave clear next steps	7	17.1
Shared knowledge about cCMV	11	26.8
Was honest about uncertainty	2	4.9
Explained what was known and what remained uncertain	1	2.4
Coordinated referrals/follow-up	2	4.9

Supplemental Materials

Provided written resources	1	2.4
Emphasized that every child with cCMV is different	1	2.4
